# The global spread of Zika virus: is public and media concern justified in regions currently unaffected?

**DOI:** 10.1186/s40249-016-0132-y

**Published:** 2016-04-19

**Authors:** Narayan Gyawali, Richard S. Bradbury, Andrew W. Taylor-Robinson

**Affiliations:** Infectious Diseases Research Group, School of Medical & Applied Sciences, Central Queensland University, Rockhampton, 4702 QLD Australia

**Keywords:** Zika, Flavivirus, Arbovirus, Mosquito, *Aedes*, Transmission, Vector control, Diagnosis, Treatment, Epidemic

## Abstract

**Background:**

Zika virus, an *Aedes* mosquito-borne flavivirus, is fast becoming a worldwide public health concern following its suspected association with over 4000 recent cases of microcephaly among newborn infants in Brazil.

**Discussion:**

Prior to its emergence in Latin America in 2015–2016, Zika was known to exist at a relatively low prevalence in parts of Africa, Asia and the Pacific islands. An extension of its apparent global dispersion may be enabled by climate conditions suitable to support the population growth of *A. aegypti* and *A. albopictus* mosquitoes over an expanding geographical range. In addition, increased globalisation continues to pose a risk for the spread of infection. Further, suspicions of alternative modes of virus transmission (sexual and vertical), if proven, provide a platform for outbreaks in mosquito non-endemic regions as well. Since a vaccine or anti-viral therapy is not yet available, current means of disease prevention involve protection from mosquito bites, excluding pregnant females from travelling to Zika-endemic territories, and practicing safe sex in those countries. Importantly, in countries where Zika is reported as endemic, caution is advised in planning to conceive a baby until such time as the apparent association between infection with the virus and microcephaly is either confirmed or refuted. The question arises as to what advice is appropriate to give in more economically developed countries distant to the current epidemic and in which Zika has not yet been reported.

**Summary:**

Despite understandable concern among the general public that has been fuelled by the media, in regions where Zika is not present, such as North America, Europe and Australia, at this time any outbreak (initiated by an infected traveler returning from an endemic area) would very probably be contained locally. Since *Aedes* spp. has very limited spatial dispersal, overlapping high population densities of mosquitoes and humans would be needed to sustain a focus of infection. However, as *A. aegypti* is distinctly anthropophilic, future control strategies for Zika should be considered in tandem with the continuing threat to human wellbeing that is presented by dengue, yellow fever and Japanese encephalitis, all of which are transmitted by the same vector species.

**Electronic supplementary material:**

The online version of this article (doi:10.1186/s40249-016-0132-y) contains supplementary material, which is available to authorized users.

## Multilingual abstracts

Please see Additional file 1 for translations of the abstract into the six official working languages of the United Nations.

## Background

The start of 2016 has been marked by global alarm over the sudden and explosive emergence of a Zika virus (ZIKV) outbreak in the majority of Latin American and Caribbean countries, with estimated cases of 440 000–1 300 000 in Brazil alone [1, 2]. The virus has been linked to more than 4 000 recent cases of microcephaly in Brazil [3], a rare congenital disease that is associated with incomplete brain development and which causes babies to be born with unusually small heads and, in the majority of cases, brain damage. It should be noted that this association, whilst compelling still requires extensive scientific study to be proven as attributable to congenital ZIKV infection. Further research is already underway in Brazil, Colombia and El Salvador to understand the effects of ZIKV in pregnant women [4].

Serological evidence has been shown recently to link ZIKV infection with another neurological disorder, Guillain-Barré syndrome (GBS), a neural demyelination disorder often identified as being an autoimmune sequela of infectious disease which causes acute or subacute flaccid paralysis [5]. After a large cluster of microcephaly and GBS was found to occur in areas newly infected with ZIKV, on February 1^st^ 2016 the International Health Regulations Emergency Committee of the World Health Organization declared ZIKV as “a public health emergency of international concern” and highlighted the importance of aggressive measures to reduce infection, particularly among pregnant women and women of childbearing age [6]. Subsequently, the US Centers for Disease Control and Prevention (CDC) have moved ZIKV to a level 1 activation [7], the highest response level at the agency. This is significant as within its three different operational areas of surveillance, diagnostics and awareness, CDC collaborates with international governments and their health agencies, industry partners at home and abroad, and with state and local health departments to strengthen response efforts to educate, alert and direct healthcare providers, media and the public about the threat posed by Zika.

This article aims to provide a balanced perspective on the possibility of a global spread of Zika, and the impact this may have on those regions currently unaffected, in light of growing public and media concern.

## Causative agent of infection and clinical manifestations of disease

Zika is a single-stranded, positive sense RNA virus [8], a member of the *Flaviviridae* family that notably also includes dengue, yellow fever and Japanese encephalitis viruses, each of which is the cause of a significant re-emerging infectious disease in humans. Zika was originally isolated in 1947 from a Rhesus macaque that was captured in the remote Zika forest of Uganda, East Africa, during the course of mosquito and primate surveillance [9]. Subsequently, the first human case was described in 1952 in Nigeria [10].

Similarly to the globally established fellow flaviviruses dengue and yellow fever, as well as the alphavirus chikungunya, ZIKV is transmitted by species of *Aedes* mosquitoes, including the widespread *A. aegypti* and *A. albopictus* [11]. Moreover, sexual contact has also been implicated as a potential mode of transmission [12, 13]. This suspicion has intensified further after the detection of ZIKV in a man in the USA who had no travel history to any Zika-endemic country but who did have a history of sexual contact with a female from a Zika-endemic region [14].

Clinically, apart from the profound effects on the unborn child apparently attributable to congenital infection, Zika infection in humans is relatively benign. In adults it is characterised as a mild, often unapparent, dengue-like disease with fever, muscle pain, conjunctivitis, eye pain, prostration and maculopapular rash [15]. In fact, around four in five adults may be infected without showing any clinical signs, in which instance they may act as asymptomatic carriers of infection for a period of several days after being bitten by an infectious mosquito [16].

## Chronology of Zika outbreaks

Based on phylogenetic analysis, the earliest date of the emergence of ZIKV is considered to be 1920 with a confidence range of 1882–1947 [17]. The enzootic cycle of ZIKV was maintained between wild primates and their vectors, possibly *Aedes* mosquitoes and occasional human infection was considered as no more than inadvertent spill over [18]. It is suspected that around 1945 the virus transferred from Africa to South East Asia, where it became geographically widespread by the 1960s [19]. In 2007, ZIKV emerged on the Micronesian island of Yap, the detection of which was suspected as an Asian lineage virus [20]. A subsequent outbreak occurred in 2013 and 2014 in French Polynesia, which was associated with 42 cases of GBS [21]. A serological finding of 0.8 % antibody positivity to ZIKV in that region suggests that the virus was not introduced prior to the outbreak [22]. A phylogenetic assessment found the Polynesian ZIKV to be very closely related to South East Asian strain. In the last year, further spread has occurred in several countries of Oceania, including New Caledonia, the Cook Islands and Easter Island [23].

From the start of 2015, patients with Zika symptoms began to be reported in the north-east of Brazil [24]. The presence of *Aedes* mosquitoes across Latin America coupled with a suitable climate for their population growth, combined with an ever increasing movement of people globally, appears to have triggered the current remarkable rise in Zika cases. Moreover, as the region is presumed to be a novel location for Zika, the inhabitants will likely not have any prior exposure to ZIKV and thus will not have ever produced specific antibodies, which act as natural vaccine against the virus. In circumstances that are distinct from the sporadic infections which have arisen within populations in Africa and Asia for several decades, in the present Zika epidemic a substantial number of people must have been affected over a short period of time in order for the outbreak to expand rapidly from its initial localized focus of infection.

**Fig. 1:**
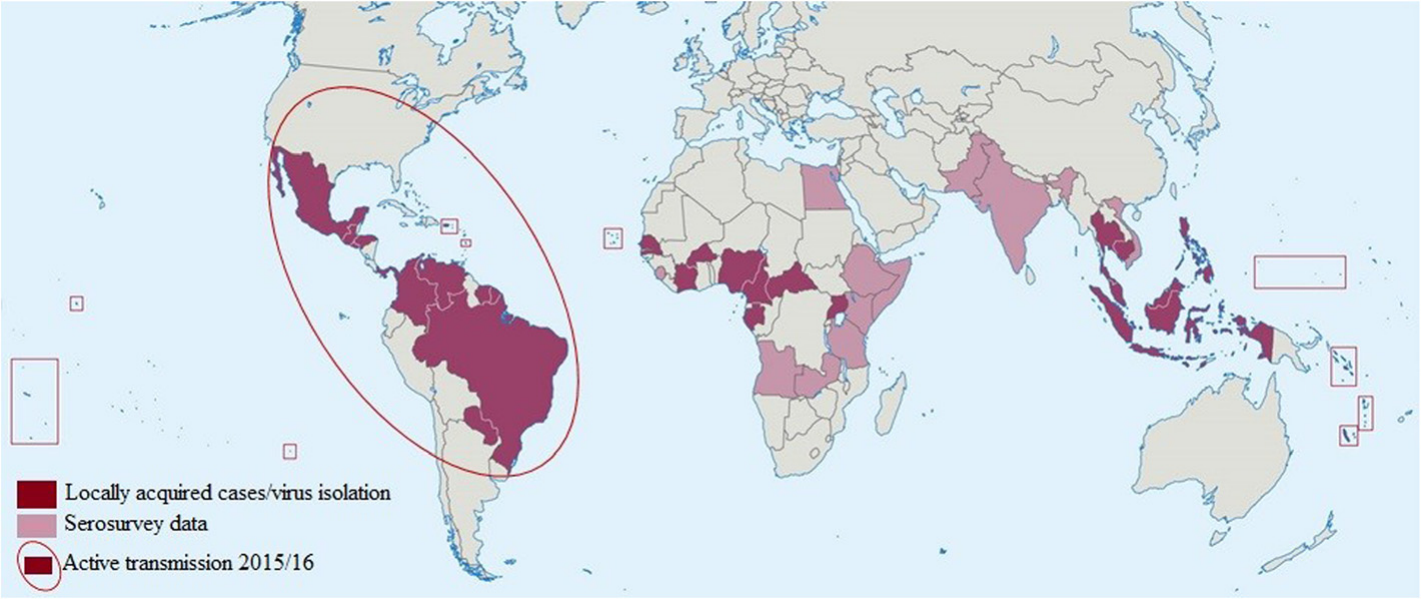
Current regions of known Zika virus endemicity [3]

By the end of 2015, ZIKV had spread to at least 18 states of Brazil. The strain of ZIKV in Latin America is thought to have originated from the Pacific islands, quite possibly brought into Brazil by one or more infected persons associated with a mass gathering such as a carnival or sporting event [1]. As an example, the international canoe race, which was held in Rio de Janeiro, Brazil, in August 2014 involved the participation of athletes from four Pacific Ocean territories (French Polynesia, New Caledonia, Cook Islands and Easter Island). Introduction by Brazilian travellers returning from the Pacific islands also cannot be excluded. By the start of March 2016, ongoing transmission of ZIKV has now been reported in 34 South and Central American countries and territories (Fig. 1). Hitherto considered for many years to be of little clinical or epidemiological importance, why this Zika epidemic has occurred now in such an explosive fashion throughout the Americas is not entirely clear, although inadequate mosquito control might be implicated as one contributing factor. In many ways, Zika is a prime example of a re-emerging infectious disease; an old disease presenting in large numbers and in a new context.

## Global spread and risk to mosquito non-endemic regions

The risk of local transmission of ZIKV and the threat of outbreaks is equal for all the countries where mosquitoes susceptible to or competent for ZIKV are found. Such species include *Aedes aegypti, A. albopictus, A. luteocephalus*, *A. unilineatus* and *A. vittatus* [25]. The potential for *A. aegypti* and *A. albopictus* - well known vectors for dengue, yellow fever and chikungunya – to transmit ZIKV is acknowledged. However, some strains within the same species may not be able to transfer ZIKV [26, 27]. Nonetheless, nations that are endemic for dengue, yellow fever and chikungunya are potentially at risk for Zika. It may be that a similar pattern of global spread could be observed when Zika, dengue and chikungunya are compared. Chikungunya was first identified in Africa before reaching South Asia, South East Asia, Pacific islands and the Americas [28]. However, no sufficiently well-defined and robust mathematical model of epidemiology applies to any communicable infectious disease [29].

Increased globalisation continues to pose a risk for disease spread. The breakdown of the destination of international travellers (9.9 million in 2015) flying from Brazilian airports (ZIKV-affected area) to North America, Europe, Asia and Africa are 65, 27, 5 and 3 %, respectively [30]. Taken together, the earlier experiences of the route of spread of dengue and chikungunya and the current data on people travelling from Brazil to overseas, point to the threat of ZIKV potentially spreading across Latin America, the Caribbean and into southern parts of the USA. By early March 2016, 153 travel-associated and 107 locally acquired Zika cases had been reported in the USA and in its territories [31]. In Asia, although there are no reports of severe outbreaks yet, sporadic cases have being described from different parts of that region [32–35]. The isolation of ZIKV from a mosquito in Malaysia in the 1960s [19] suggests that the virus has existed there for a long time and so may have caused morbidity for many years, probably being underreported due to confusion of diagnosis with dengue fever, pyrexia of unknown origin or other unknown diseases. Serological or phylogenetic studies are required in order to determine the extent of the spread of ZIKV in regions of South Asia, where dengue already has a strong presence. It may be possible that people in such areas could have developed antibodies through infection with ZIKV over an extended period, which could provide a naturally acquired form of prophylaxis. This possibility lowers the assumed risk of the immediate spread and outbreaks of Zika in tropical parts of Asia.

In addition to the spread of Zika via mosquito bite, the current news media is awash with stories regarding the possibilities of sexual, blood-borne and vertical transmission of ZIKV. In a scenario in which one or more of these routes proves to be a potential mode of transmission, paradoxically the effect may be to facilitate any outbreak of Zika in those areas regardless of *Aedes* mosquito availability. This would pose a serious threat for the global spread of ZIKV and thus constitutes a major public health problem.

## Limited risk of local transmission in currently unaffected regions

While it is possible that the distribution of Zika in the Asia Pacific region could eventually reach as far as Australia, it should be containable in this and other developed countries where *Aedes* spp. mosquitoes are found through adherence to current measures of mosquito control. Such vector control programs are in place to combat dengue, yellow fever and Japanese encephalitis. It is highly likely that any outbreak initiated by an infected index case returning from overseas would be locally restricted, just like current dengue outbreaks that occur sporadically in northern Queensland [36]. As *A. aegypti* has a very limited flight range [37], to sustain a significant outbreak requires a high population density of both mosquitoes and humans. In this context, it should be noted that this is a mosquito species that thrives in urban environments which is a consideration for future control programs, both for Zika itself but also in unison with the ongoing public health threat posed by dengue and other medically important arboviruses.

## Strategies to combat ZIKV

There is no vaccine available to immunize people against Zika. Since ZIKV is a relative of yellow fever and Japanese encephalitis viruses, for which efficacious vaccines are available [38], there is hope that a similar preparation can be developed against Zika but this may take several years to design, trial and gain regulatory approval for public administration [39]. With anti-viral therapy also not available, at present alternative interventions are being used to combat Zika. These include low technology measures that focus on vector control such as insecticide spraying, limit mosquito breeding and providing protection from mosquito bites [40]. *Aedes* mosquitoes are active and bite during the day [41], so use of an effective repellent is highly recommended. The fitting of house door and window screens, use of air-conditioning, removal of yard and household debris and containers (e.g. disposed tyres, broken bowls and cups, flower vases) that provide breeding sites for mosquitoes, are all measures that contribute to control of the vector within a local community [40].

Integrated vector management (IVM), advocated by the World Health Organization as a method of combating *Aedes* transmission of dengue [42], would also be an appropriate strategy for ZIKV. IVM is defined as “a rational decision-making process for the optimal use of resources for vector control”, where an integrated collaborative sector implements evidence-based selection and delivery of different interventions (or combinations of interventions) that are informed by, and thereby tailored to, local settings [42]. Until such time as the apparent association between ZIKV infection and microcephaly is either established or disproved, women should be cautious in planning to conceive a baby or to travel to a Zika-endemic country if already pregnant. An effective approach to surveillance of infection among pregnant women, at least in current endemic regions, should be initiated [43]. While the mooted possibility of sexual and vertical transmission of ZIKV remains to be substantiated [13], practicing safe sex in Zika ‘hot spots’ would beneficially reinforce existing protection programs against sexually transmitted diseases including HIV/AIDS. The real possibility of ZIKV transmission through blood transfusion should not be ignored [44]. Thus, if not already in place, a stringent safety policy for managing blood and blood products should be established and practiced.

## Research priorities to prevent a ZIKV pandemic

Given the magnitude of the current explosive spread of Zika [1, 2], public and private key stakeholders, funding agencies and public health experts worldwide should consider what issues need to be prioritised for research in order to produce effective approaches to combat ZIKV. Very little is known about this virus [45]; the shortfall in research includes epidemiological characteristics, surveillance and diagnostics, virus reservoirs/vectors/transmission, disease manifestations and sequelae, clinical management and public health interventions. The drivers responsible for Zika emergence and outbreak are evidently complex and multifactorial [45]. This collective knowledge gap demands urgent action to characterise better both the virus and the pathologies it causes, especially those related to infection during pregnancy, which represents a serious public health challenge. Beyond short term emergency management of the Latin American epidemic [6, 7], a major consideration is to research strategies for prevention of a global pandemic, including effective vaccine development and implementation of vector control programs.

## Conclusions

The recent striking emergence of cases of Zika in Latin America poses a threat for a worldwide outbreak of this mosquito-borne viral infection. The globalisation of the human population promotes the movement of asymptomatic carriers between countries in the same or different continents. Pregnant women are considered a high-risk group for Zika, as infection is strongly associated with, if as yet unproven as a cause of, microcephaly in the developing foetus. Furthermore, increasing suspicion of diverse modes of virus transmission, sexual and vertical, if confirmed, makes the life cycle of the virus extremely complex and limits the scope of predictive models, which in turn may impede control efforts. No vaccine has been developed yet and even though this has been prioritised by multiple funding agencies, it may take several years to become commercially available. In this context, performing clinical trials on pregnant women would present difficult practical and ethical hurdles to overcome.

Overall, the public health challenges presented by ZIKV raise a significant threat for a global outbreak unless and until the current deficit of knowledge relating to the epidemiology of this virus is rectified. In more economically developed countries, however, Zika may not have the same impact as it is making in regions in which there is insufficient funding or infrastructure to support effective implementation of mosquito control programs. In this regard, it would be appropriate to consider Zika as an infectious disease of poverty. ZIKV may spread to countries adjoining those now heavily affected and small scale, easily controlled, outbreaks may occur through travel in industrialised nations. However, given the significant capacity for mosquito control in developed countries, we consider that the widespread public and media concern regarding the spread of Zika to, and potential to reach epidemic proportions in, currently unaffected industrialised nations as difficult to justify.

### Ethics approval and consent to participate

Not applicable.

### Consent for publication

Agree to publish.

### Availability of data and materials

Not applicable.

## Additional file

Additional file 1: Multilingual abstracts in the six official working languages of the United Nations. (PDF 334 kb)

## Abbreviations

GBS: Guillain-Barré syndrome
IVM: integrated vector management
ZIKV: Zika virus
