# Supplementary material for: The global spread of Zika virus: is public and media concern justified in regions currently unaffected?
Source: Infect Dis Poverty. 2016 Apr 19;5:37. doi: 10.1186/s40249-016-0132-y (PMC4837632; doi:10.1186/s40249-016-0132-y)
Supplement: Additional file 1: — Multilingual abstracts in the six official working languages of the United Nations. (PDF 334 kb) [file 40249_2016_132_MOESM1_ESM.pdf]

Translation of the abstract into the five official working languages of the United Nations

الإنتشار العالمي لفايروس زيكا: هل يعتبر القلق العالمي و الإعلامي مبرراً في المناطق الغير متضررة حالياً؟

نارايان غياوالي، ريتشارد برادبري وأندرو دبليو تايلور روبنسون

ملخص

خلفية: فايروس زيكا، هو عدوى حمى صفراء تنتقل عن طريق البعوض وسرعان ما تحولت إلى مصدر قلق صحي عالمي بعد ما اشتبه بإرتباطها ب 4000 حالة من الصعل بين الاطفال حديثي الولادة في البرازيل.

نقاش: قبل ظهوره في امريكا اللاتينية في فترة 2015 -16 كان فايروس زيكا معروف بانتشاره المحدود نسسبياً في اجزاء من أفريقيا و آسيا وجزر المحيط الهادئ. من المرجح أن السبب في انتشاره العالمي هو تغيير الظروف المناخية حيث ساهمت الظروف في نمو انواع من البعوض هي الزاعجة المصرية و الزاعجة المنقطة بالأبيض ضمن نطاق جغرافي آخذ بالتوسع. بالإضافة لذلك، تشكل العولمة المتزايدة عاملاً مساهماً في زيادة خطر انتشار العدوى. علاوة على ذلك فمن المشتبه أن هناك طرق بديلة أيضاً لانتقال العدوى (كالإنتقال الجنسي أو العمودي)، في حال ثبتت الشبهات فإن ذلك يعني عاملاً آخر لانتشار العدوى في المناطق الغير متضررة حالياً. بما أنه لم يتم التوصل لأي لقاح او مضاد للفيروسات حتى الآن فإن الطريقة الوحيدة حالياً لمنع انتشار الفايروس هو تجنب لدغات البعوض، منع النساء الحوامل من السفر إلى المناطق الموبوءة بفايروس زيكا و زيادة التوعية حول طرق الإتصال الجنسي الآمن في تلك المناطق. الأهم من ذلك أن يتم توخي الحذر في تلك المناطق في حال التخطيط لإنجاب أطفال إلى أن يتم تأكيد أو نفي الصلة بين الإصابة بفايروس زيكا و بين حالات الصعل. السؤال الذي يطرح نفسه الآن هو حول النصيحة المناسبة التي يمكن أن تُقدّم للدول المتقدمة صناعياً البعيدة عن المناطق الموبوءة و التي لم يتم الإبلاغ فيها عن وجود فايروس زيكا.

خلاصة القول: على الرغم من القلق المبرر والذي تم تغذيته من قبل وسائل الإعلام والذي انتشر بين عامة الناس في المناطق الغير موبوءة مثل أمريكا الشمالية، أوروبا و استراليا، فإنه من الممكن حالياً إحتواء أي عدوى على الصعيد المحلي (انتقلت عن طريق أحد المسافرين من المناطق الموبوءة).
بما أن اصناف البعوض تتميز بانتشار مكاني محدود جداً فيجب أن يكون هناك تداخل بين كثافة سكانية بشرية عالية و مجموعات كبيرة من البعوض لكي تتشكل بؤرة عدوى. لكن بما أن بعوض الزاعجة المصرية معروف بولعه بالبشر فإنه من الواجب دراسة استراتيجيات مستقبلية لمراقبة فايروس زيكا بجانب التهديد المستمر لصحة البشر الذي يتمثل بحمى الضنك، الحمى الصفراء و التهاب الدماغ الياباني والتي جميعها تنتقل عن طريق انواع حشرات ناقلة متشابهة

Translated from English version into Arabic by Rola Majbor, through

TRANSLATORS
WITHOUT BORDERS

寨卡病毒全球传播：尚未被波及地区的公众和媒体的关注合理吗？

Narayan Gyawali, Richard S. Bradbury and Andrew W. Taylor-Robinson

摘要：

引言：寨卡病毒，一种伊蚊传播的黄病毒，因为怀疑与近来巴西 4000 多例新生儿头小畸形有关，因而迅速成为全球公共卫生的焦点。

讨论：在 2015-2016 年拉美的暴发之前，寨卡病毒只在非洲、亚洲和太平洋岛国部分地区低水平流行。此次明显的全球播散可能因气候变化从而适宜埃及伊蚊和白纹伊蚊在全球更大范围地繁殖有关。此外，不断加剧的全球化持续导致感染扩散风险。而且，可疑存在其他的病毒传播模式（性传播和垂直传播），如果被证实，那么也为蚊虫非流行区的暴发提供了平台。因为目前尚没有疫苗和抗病毒治疗方法，当前预防措施是保护免受蚊虫叮咬、孕妇不要前往寨卡病毒流行区旅行、进行安全的性行为。尤其是在有寨卡病毒流行

的国家，建议暂缓怀孕直到寨卡病毒感染与头小畸形的关系被证实或否定。问题是该为那些远离流行区的经济发达国家和尚无寨卡病毒感染报告的国家提供什么样的建议合适呢。

总结：在北美、欧洲和澳洲尚无寨卡病毒出现的地区，尽管公众受到媒体刺激从而对此极为关注可以理解，但是目前任何暴发（前往流行区的旅游者回国引发）都非常有可能被在当地遏制。因为伊蚊空间分布有限，需要高密度蚊媒和人群的重叠才能支撑疫源地形成。但是，因为埃及伊蚊明确的嗜人特性，未来的寨卡病毒控制策略需要考虑与那些对人类健康构成持续威胁的疾病如登革、黄热病和日本脑炎的整合，它们都由相同的媒介传播。

Translated from English version into Chinese by Men-Bao Qian, through

CHINESE CENTER FOR DISEASE CONTROL AND PREVENTION
NATIONAL INSTITUTE OF PARASITIC DISEASES

La propagation mondiale du virus Zika : les préoccupations du public et des médias sont-elles justifiées dans les régions jusqu’à présent épargnées ?

Narayan Gyawali, Richard S. Bradbury et Andrew W. Taylor-Robinson

Résumé

Contexte : le virus Zika, un flavivirus transmis par les moustiques *Aedes*, est rapidement devenu un problème de santé publique suite à son association supposée avec plus de 4000 cas récents de microcéphalie affectant des nouveau-nés au Brésil.

Discussion : préalablement à son apparition en Amérique du Sud en 2015-16, la prévalence de Zika à un taux relativement faible était connue dans certaines régions d’Afrique, d’Asie et des îles du Pacifique. Une extension de sa dispersion mondiale manifeste peut être permise par des conditions climatiques favorables à la croissance de la population de moustiques *A. aegypti* et *A. albopictus* sur une zone géographique en extension. De plus, la mondialisation toujours plus importante continue de constituer un risque de propagation de l’infection. Les modes alternatifs de transmission du virus (sexuelle et verticale) soupçonnés peuvent, si leur existence est prouvée, fournir une plate-forme de propagation à des régions où ces moustiques ne sont pas endémiques. Étant donné l’absence d’un vaccin ou d’un traitement antiviral, les moyens actuels de prévention de la maladie impliquent la protection contre les piqûres de moustique, l’interdiction aux femmes enceintes de se rendre dans des zones où Zika est endémique et l’adoption de pratiques sexuelles sans risque dans ces pays. Il est important d’apporter une précaution particulière à la planification de la conception d’un bébé dans les pays où Zika est endémique, étant donné que l’association manifeste entre l’infection par le virus et la microcéphalie n’est ni confirmée, ni réfutée. La question se pose de savoir quels conseils il convient de donner dans les pays plus développés d’un point de vue économique et étant éloignés de l’épidémie actuelle où Zika n’a pas encore été signalé.

Résumé : malgré les préoccupations compréhensibles exprimées par le public et ayant été alimentées par les médias dans les régions où Zika n’est pas présent, comme l’Amérique du Nord, l’Europe et l’Australie, il y a actuellement toutes les chances pour qu’une épidémie (causée par un voyageur infecté revenant d’une zone endémique) soit contenue à un niveau local. Les espèces *Aedes* présentent une dispersion spatiale très limitée et le recoupement de fortes densités de population de moustiques et d’êtres humains serait nécessaire à la persistance d’un foyer d’infection. Cependant, le moustique *A. aegypti* étant distinctement antropophilique, il convient d’envisager de futures stratégies de lutte contre Zika en association avec la menace permanente à l’égard du

bien-être humain représentée par la dengue, la fièvre jaune et l'encéphalite japonaise qui sont toutes transmises par les mêmes espèces vectrices.

Translated from English version into French by eric ragu, through

Глобальное распространение вируса Зика: обосновано ли беспокойство населения и средств массовой информации в регионах, не затронутых инфекцией в настоящий момент?

Нараян Гиавали, Ричард С. Брэдбери и Эндрю В. Тэйлор-Робинсон

Резюме

Предпосылки: Переносимый комарами рода *Aedes* флавивирус, известный как вирус Зика, быстро стал поводом для беспокойства о здоровье населения в связи с предположением, что он может являться причиной более 4000 недавних случаев микроцефалии у новорожденных в Бразилии.

Анализ ситуации: До своего прихода в Латинскую Америку в 2015-2016 годах вирус Зика существовал в отдельных регионах Африки, Азии и на тихоокеанских островах. Нарастающие темпы глобального распространения вируса могут быть связаны с климатическими условиями, благоприятными для увеличения территории размножения желтолихорадочных и азиатских тигровых комаров (*A. aegypti* и *A. albopictus*), а в связи с расширением глобализации риск распространения инфекции еще больше увеличивается. Кроме того, в случае подтверждения альтернативных путей передачи вируса, таких как половой и вертикальный, в зону риска также войдут регионы, не населенные комарами. Поскольку вакцины или антивирусной терапии пока не существует, текущими методами предотвращения инфекции являются защита от комариных укусов, запрещение беременным женщинам посещать регионы, в которых распространен вирус Зика, а также практика безопасного секса в этих странах. Так, в странах, где вирус Зика носит эндемический характер, рекомендуется воздержаться от планирования зачать ребенка, до того как возможная связь между вирусной инфекцией и микроцефалией подтвердится или, наоборот, опровергнется. Также встает вопрос, что можно посоветовать более экономически развитым странам, удаленным от очага эпидемии, в которых вирус Зика пока не обнаружен.

Заключение: Несмотря на понятную тревогу среди населения, усиленную средствами массовой информации, в таких регионах, как Северная Америка, Европа и Австралия, где вирус Зика не зафиксирован, в настоящий момент любая вспышка инфекции, спровоцированная зараженным человеком, посетившим эндемичный регион, с большой вероятностью будет погашена на местном уровне. Зона перемещения комаров *Aedes* весьма ограничена, для подтверждения очага инфекции необходимо пересечение высоких плотностей обитания комаров и людей. Но поскольку желтолихорадочные комары *A. aegypti* антропофильны, будущие стратегии контроля вируса Зика должны учитывать постоянную угрозу здоровью человека, которую представляют лихорадка денге, желтая лихорадка и японский энцефалит, распространяемые этими видами-переносчиками.

Translated from English version into Russian by Elena Surina, through

La propagación mundial del virus del Zika: ¿se justifica la preocupación del público y de los medios en regiones actualmente no afectadas?

Narayan Gyawali, Richard S. Bradbury y Andrew W. Taylor-Robinson

Resumen

Antecedentes: El virus del Zika, un flavivirus transmitido por el mosquito *Aedes*, se está convirtiendo rápidamente en una preocupación mundial en el ámbito de la salud pública luego de su supuesta vinculación con más de 4.000 casos recientes de microcefalia en bebés recién nacidos en Brasil.

Discusión: Previo a su emergencia en Latinoamérica en los años 2015-16, se sabía que el Zika tenía una prevalencia relativamente baja en ciertas regiones de África, Asia y las islas del Pacífico. Una extensión de su aparente diseminación a nivel global pudo haber sido permitida por condiciones climáticas favorables para el crecimiento de la población de mosquitos *A. aegypti* y *A. albopictus* en una zona geográfica que se extiende cada vez más. Asimismo, la creciente globalización continúa siendo un riesgo para la propagación de la infección. Más aún, la sospecha de modos alternativos de transmisión del virus (sexual y vertical), de comprobarse, ofrecen asimismo una plataforma para brotes en regiones no endémicas para el mosquito. Debido a que todavía no existe una vacuna o terapia antiviral, los medios actuales de prevención de la enfermedad involucran la protección contra picaduras de mosquito, evitar que mujeres embarazadas viajen desde zonas endémicas para el Zika, y la práctica de sexo seguro en dichos países. Es importante que en los países donde el Zika se reporta como endémico, se recomiende tomar precaución en la planificación para la concepción de bebés hasta que la posible relación entre la infección por el virus y la microcefalia se confirme o se desmienta. La pregunta que surge es qué consejo resulta el adecuado para aquellos países económicamente más desarrollados que se encuentran lejos de la epidemia actual y en los cuales todavía no se ha reportado el Zika.

Resumen: A pesar de la inquietud entendible entre el público en general propulsada por los medios, en regiones donde el Zika no está presente, como América del Norte, Europa y Australia, en estos momentos cualquier brote (iniciado por un viajero infectado que regresa de una zona endémica) muy probablemente será contenido de manera local. Debido a que *Aedes* spp posee una dispersión espacial muy limitada, sería necesaria la superposición de una alta densidad de población de mosquitos con una de seres humanos para mantener un foco de infección. Sin embargo, como *A. aegypti* es claramente antropofílico, deberían considerarse futuras estrategias de control para el Zika en conjunto con la amenaza continua al bienestar de la población humana que presentan el dengue, la fiebre amarilla y la encefalitis japonesa, todas transmitidas por la misma especie de vector.

Translated from English version into Spanish by Maria Alejandra Aguada, through
